# Supplementary material for: Predicting visual function by interpreting a neuronal wiring diagram
Source: Nature. 2024 Oct 2;634(8032):113–23. doi: 10.1038/s41586-024-07953-5 (PMC11446822; doi:10.1038/s41586-024-07953-5)
Supplement: Supplementary file 6 — CRF and ERF predictions for individual TmY4 and TmY9 cells. Analogous to Supplementary Data 3, but for TmY target types. Shown are the top four monosynaptic pathways, the strongest pathway passing through each of the top ten intermediary types (ranking from Extended Data Fig. 7), and the trisynaptic pathway Tm1–TmY–Dm3–TmY (see the section entitled Prediction of spatial normalization). [file 41586_2024_7953_MOESM6_ESM.zip › DataS4/TmY9q/720575940624755896.pdf]

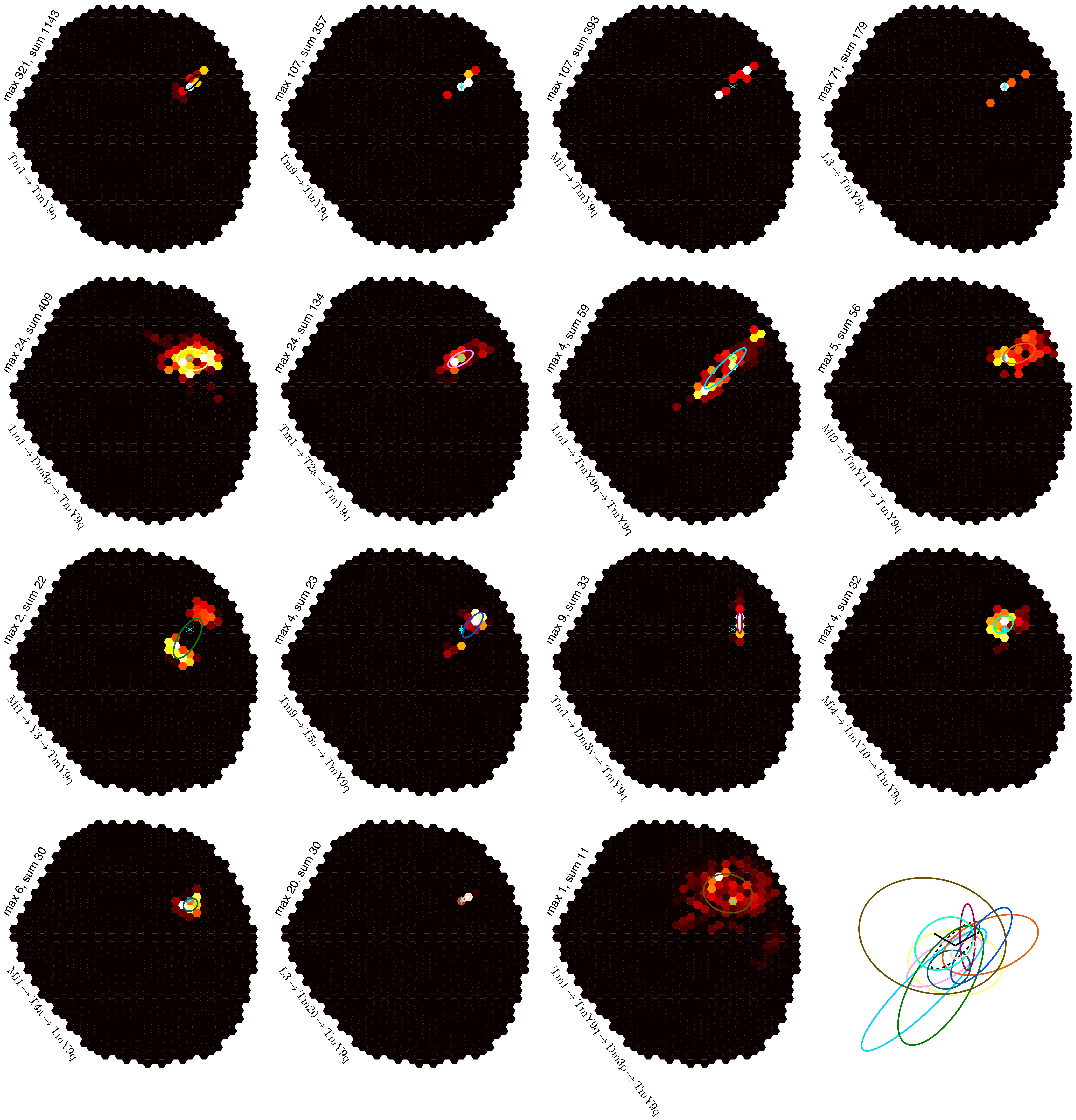
max 321, sum 1143
Tm1 → TmY9q
max 107, sum 357
Tm9 → TmY9q
max 107, sum 393
Mi1 → TmY9q
max 71, sum 179
L3 → TmY9q
max 24, sum 409
Tm1 → Dm3p → TmY9q
max 24, sum 134
Tm1 → T2a → TmY9q
max 4, sum 59
Tm1 → TmY9q → TmY9q
max 5, sum 56
Mi9 → TmY11 → TmY9q
max 2, sum 22
Mi1 → Y3 → TmY9q
max 4, sum 23
Tm9 → T5a → TmY9q
max 9, sum 33
Tm1 → Dm3v → TmY9q
max 4, sum 32
Mi4 → TmY10 → TmY9q
max 6, sum 30
Mi1 → T4a → TmY9q
max 20, sum 30
L3 → Tm20 → TmY9q
max 1, sum 11
Tm1 → TmY9q → Dm3p → TmY9q
